# Differential Outcomes Training Ameliorates Visual Memory Impairments in Patients With Alzheimer’s Disease: A Pilot Study

**DOI:** 10.3389/fpsyg.2018.02671

**Published:** 2019-01-11

**Authors:** Isabel Carmona, Ana B. Vivas, Angeles F. Estévez

**Affiliations:** ^1^ Department of Psychology, University of Almería, Almería, Spain; ^2^ Department of Psychology, University of Sheffield International Faculty, CITY College, Thessaloniki, Greece; ^3^ CERNEP Research Center, University of Almería, Almería, Spain

**Keywords:** Alzheimer’s disease, differential outcomes procedure, visual recognition memory, long-term retention, cognitive training

## Abstract

It is well known that Alzheimer’s disease (AD), the most common form of dementia, is associated with deficits in cognitive processes including visual memory impairments. One technique that might be used to ameliorate these impairments is the differential outcomes procedure (DOP) that involves associating each to-be-remembered stimulus with a specific outcome.

**Objective:** Previous research has demonstrated that the DOP can be used to reduce or eliminate the learning and memory deficits associated with animal models of amnesia and dementia. Furthermore, this procedure has been shown to improve delayed facial recognition in healthy older adults as well as in patients diagnosed with AD. The main aim of the present study is twofold: to extend these findings to other types of visual stimulus and to investigate the effect of the DOP in memory retention in AD patients.

**Method:** Ten patients diagnosed with AD and 10 healthy controls participated in this study. The experiment included two phases. In the first one, they had to perform a delayed matching-to-sample task. In the second phase, participants performed a recognition memory task, designed to assess long-term retention, 1 h and 1 week after the training.

**Results:** Participants showed a better memory-based performance as well as a higher long-term retention of the information when trained under the differential outcomes condition, relative to the non-differential outcomes condition.

**Conclusions:** The DOP seems to be an effective, easy-to-implement, technique to enhance visual memory in AD patients.

## Introduction

According to the World Health Organization (2017), 50 million people in the world suffer from dementia, with nearly 10 million new cases every year. It is also reported that Alzheimer’s disease (AD) accounts for 60–70% of dementia cases worldwide. Consequently, AD stands as a major public health priority, which has led to considerable research efforts in early diagnosis, prevention, and intervention programs to palliate the devastating effects of the disease. In the early stages of AD, the main cognitive processes affected are memory and executive functions such as planning, decision-making, or reasoning (McKhann et al., 2011). These first signs are followed by the gradual deterioration of other cognitive functions, along with a significant decline in everyday functions. It has also been suggested that visual memory deficits are most significant in AD and are the best early predictors of clinical onset (Kawas et al., 2003). However, not all memory processes are affected in AD; while declarative (explicit or conscious) memory is compromised early on (Squire and Zola, 1996), non-declarative (implicit or unconscious) memory is relatively unaffected until very advanced stages of the disease (e.g., Jelicic et al., 1995; Fleischman and Gabrieli, 1998).

In the last two decades, a different line of research has shown that the manipulation of outcomes arrangements (differential vs. non-differential) after correct responses, in discriminative learning and visuospatial recognition memory, has a significant effect in the performance of diverse populations (for a review, see Mok et al., 2010; López-Crespo and Estévez, 2013). That is, the unique paring of outcomes with the to-be-learned or to-be-remembered stimulus appears to improve the learning rate of conditional symbolic relationships and accuracy and/or latency performance in memory tasks. This procedure has been termed the differential outcomes procedure (DOP). To illustrate this procedure, one can imagine a real-life situation where a patient has to learn to take different types of medications at different times of a day. In the DOP condition, patients would receive a hug from a significant other every time they correctly take the pill for arthritis in the morning; whereas they would be praised with the comment “well done” every time they correctly take the pill for hypertension at mid-day. This apparently simple way of arranging the outcomes (e.g., hug or praise) has shown to enhance memory as well as discriminative learning and long-term retention of the learned information as compared to a condition where the outcomes are randomly administrated after each correct response (e.g., Hochhalter et al., 2000; Miller et al., 2002; López-Crespo et al., 2009; Mok et al., 2009; Plaza et al., 2011; Molina et al., 2015).

Relevant to the cognitive theories of AD, it has been proposed that the positive effects of the DOP are best explained in terms of specific activation of the implicit prospective memory system. According to the two-memory system model (e.g., Savage, 2001; Savage et al., 2004; Ramirez and Savage, 2007; Savage and Ramos, 2009), the differential and non-differential outcomes procedures (DOP and NOP) involve distinctively prospective and retrospective memory, respectively. In the DOP condition, an expectancy of the outcome, a prospective process, is activated and used as an additional source of information to guide behavior choices. These expectancies are formed *via* classical conditioning associations (i.e., sample stimulus-outcome) in such a way that after several pairings the presentation of the target stimulus activates the representation of its own and unique outcome. This is an unintentional process characteristic of implicit memory, a type of memory that is relatively unaffected in patients with AD (Lusting and Buckner, 2004). By contrast, when the outcomes are randomly administered or are common to all stimuli (the NOP condition), a retrospective process is activated. This retrospective process maintains active, over the delay, the representation of the stimulus presented. Results from previous studies indicate that the prospective and retrospective memory systems recruit distinct neurobiological mechanisms in animals (e.g., Savage, 2001) as well as in humans (Mok et al., 2009; Mok, 2012). For instance, Mok and colleagues found neuroanatomical evidence, using fMRI, in support of the *two-memory system model*: the activations found in the angular gyrus (posterior parietal cortex) versus the hippocampus (medial temporal lobe) were related to the prospective (DOP condition) and the retrospective (NOP condition) memory processes, respectively. Given that, as previously mentioned, prospective implicit memory is relatively unaffected in AD patients, the DOP may enhance memory *via* this system. This hypothesis seems to be supported by a recent study on delayed face recognition using the DOP in a group of patients diagnosed with AD (Plaza et al., 2012). In this study, participants showed significantly better face recognition when differential outcomes were arranged. In fact, the performance of AD patients was equivalent to that of the control group in the shorter delay (5 s) under the DOP condition.

The aim of the present study is to further investigate the potential benefits of the DOP to improve visual recognition memory (VRM) for non-facial visual stimuli in AD patients. Although previous research has shown better face recognition in AD patients with this procedure (Plaza et al., 2012), faces differ from other visual stimuli in terms of underlying neurocognitive mechanisms (Dailey and Cottrell, 1999; Jorge et al., 2018). Therefore, it is important to demonstrate that the DOP improves recognition memory for all visual stimuli and not only for human faces. In addition, Plaza et al. (2012) did not investigate long-term effects, and so in the present study, we also measured long-term memory retention at 1-h and 1-week follow-ups. Based on previous studies, we expect to find a worst overall recognition memory performance in the AD group relative to the control group. Furthermore, we hypothesize that the group of patients will show a better memory-based performance in the DOP condition relative to the NOP condition, both during the training phase and the follow-up retention test.

## Materials and Methods

### Participants

Ten patients diagnosed with AD and nine healthy controls (HCs), who were recruited from the nursing home “Virgen de la Esperanza,” participated in the study. The exclusion criteria for HC were: 1) a serious medical condition (i.e., heart disease, cancer, stroke); 2) a history of drug and alcohol abuse; and 3) a score of 23 or less in the Mini-Mental State Examination (MMSE). The AD participants were residents of two nursing homes located in Almería (“Residencia Virgen de la Esperanza”) and in Cuevas del Almanzora, Almería (“Residencia Santa Luisa de Marillac”) and of the CEDAEN (Centre of Dementia, Alzheimer Disease and Neurodegenerative Disease of Chirivel, Almería).

The diagnosis of AD was determined by a neurologist from the Andalusian Health Service according to the National Institute of Neurological and Communicative Disorders and Stroke (NINCDS) and Alzheimer’s Disease and Related Disorders Association (ADRDA) (McKhann et al., 1984, 2011) and included neurological and neuroimaging examination and neuropsychological/neuropsychiatric assessment. Only patients in Phase 4 of the Reisberg’s Global Deterioration Scale (Reisberg et al., 1982) were included in the study. Demographic and clinical information can be found in Table 1. AD patients and HCs were matched for age and education, and all of them had normal or corrected-to-normal vision. Informed written consent to participate in the study was obtained from the participants and patient’s caregivers. The study was approved by the Human Research Ethics Committee of the University of Almería, and it was conducted in accordance with the Declaration of Helsinki.

**Table 1:** Socio demographic and clinical information for both groups (AD and HC; standard deviation in brackets).

	AD	HC
N	10	9
Sex (M/F)	(2/8)	(1/8)
Age in years	81 (7)	81 (6)
Education in years	8 (3)	8 (3)
MMSE^a^	17 (2)	28 (1)
GDS	4	-

Note: Values in parentheses are SD. MMSE = Mini-Mental State Examination. GDS = Global Deterioration Scale.

^a^ Two-sample t-test <0.001.

### Stimuli and Materials

The stimuli were two sets of four photographs of daily objects taken from the Bank of Standardized Stimuli (BOSS; Brodeur et al., 2010, 2014). All stimuli were presented on a black background on a tactile screen (15 inch, LCD monitor). The photographs measured 6.5 × 6.5 cm and could be displayed either individually at the center of the screen (sample stimulus) or in a 2 × 2 grid (comparison stimuli) equidistant from the center. The position of the photographs on the 2 × 2 grid was randomly arranged. Stimulus presentation and data collection were controlled by the E-Prime v. 2.0 Software (Psychology Software Tools, 2007).

Two sets of two photographs of natural landscapes along with two phrases (e.g., “Very good!” and “Well done!”) were used as immediate reinforces, that is as outcomes for correct responses (see Molina et al., 2015 with young adults; Plaza et al., 2018 with older adults).

Finally, two new sets of six photographs of daily objects served as novel stimuli in the long-term memory test.

### Procedure

Participants were tested individually in a quiet room. The experiment included two phases. In the first training phase, they had to perform a delayed matching-to-sample task. In the second follow-up phase (1 h and 1 week after the training), participants performed a recognition memory task designed to assess long-term retention. In this task, participants were required to make an old/new judgment; that is, they had to indicate whether the stimulus had been previously presented in phase 1.

A mixed design was used in the present study. Therefore, two versions of a delayed matching-to-sample task (the VRM tasks A and B) were designed by using two different sets of four everyday objects as stimuli. For each version, two of the four stimuli were presented as the initial cue (or sample) stimuli and the rest as the comparison stimuli. In one version, the outcomes were randomly given after each correct response (NOP task condition), whereas in the other version a unique outcome followed correct response to a particular comparison stimulus (DOP task condition). The two task conditions were run in two different sessions, 1 week apart, to prevent fatigue effects. The order of the task condition (DOP and NOP) was counterbalanced across participants. We also orthogonally combined the two stimuli sets with the two task conditions to avoid any potential bias. Thus, participants performed the task under one training condition (e.g., differential) with one set of stimuli, and a week later, they performed the task under the other training condition (e.g., non-differential) using the other set of daily objects.

**Phase 1 (training). Delayed matching to sample task**. The instructions for the task were provided both in written and orally. After the instructions, each participant was required to make a practice block of four trials to ensure correct understanding of the task. These practice trials were identical to the training trials.

**Figure 1:**
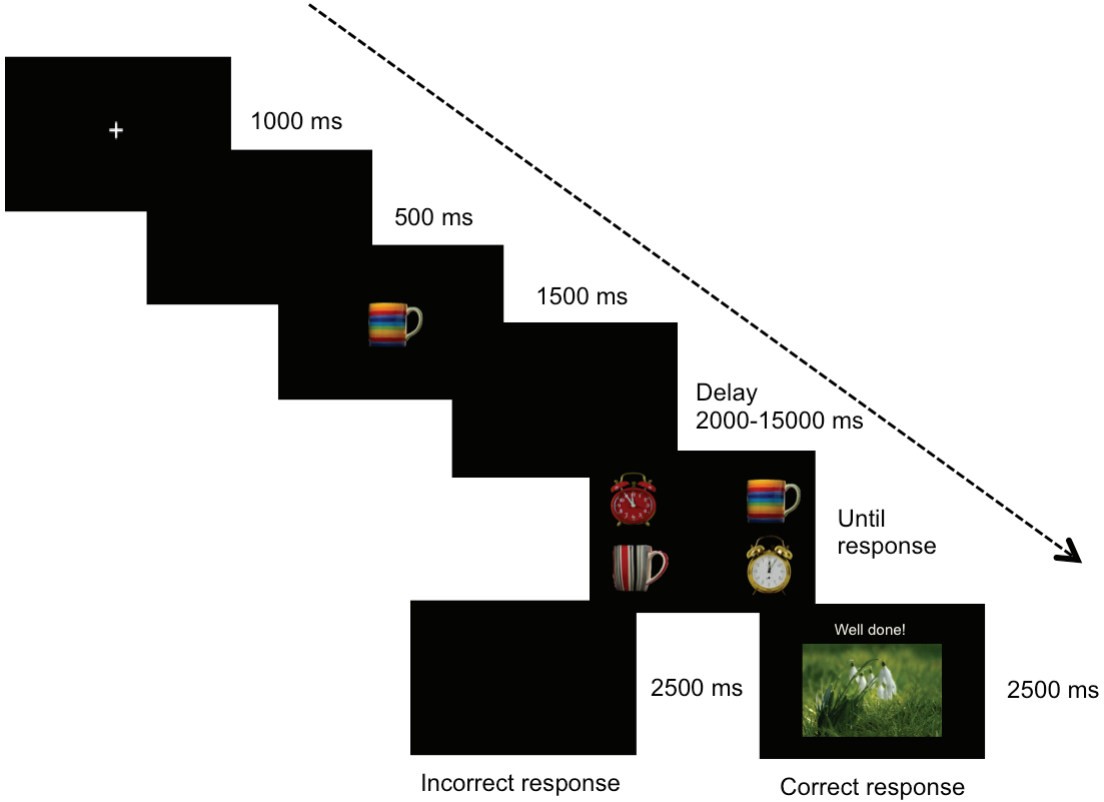
Stimuli sequence (from left to right). The photographs included in this figure are public domain images obtained from Pixabay (https://pixabay.com/; Creative Commons License, no attribution required).

The experimental task consisted of a delayed matching-to-sample task with 36 training trials grouped in three blocks of 12 trials each. Each trial (see Figure 1) began with a fixation cross presented for 1,000 ms followed by an interval of 500 ms with a blank screen. Then, a photograph of a daily object (the cue stimulus) was presented in the middle of the screen for 1,500 ms. Each sample stimulus was repeated six times per block. Thus, each cue stimulus was presented 18 times as a sample stimulus and 36 times as a comparison stimulus. After a varying delay of 2 or 15 s (randomly selected) in which the screen remained blank, four comparison stimuli were presented. Participants had to select the sample stimulus by touching the screen. The comparison stimuli lasted until the participants responded or during 10 s. Correct responses were then followed by the outcome for 2,500 ms. Incorrect responses were followed by a blank screen for 2,500 ms. Then, the next trial began.

In the DOP condition, each sample stimulus was always associated with a unique outcome (a particular photograph of a natural landscape paired with a particular congratulation phrase), and each correct response to this stimulus was followed only by that pair outcome. By contrast, in the NOP condition, correct responses were followed by a randomly selected pair outcome, which again consisted of a photograph and a congratulation phrase.

**Figure 2:**
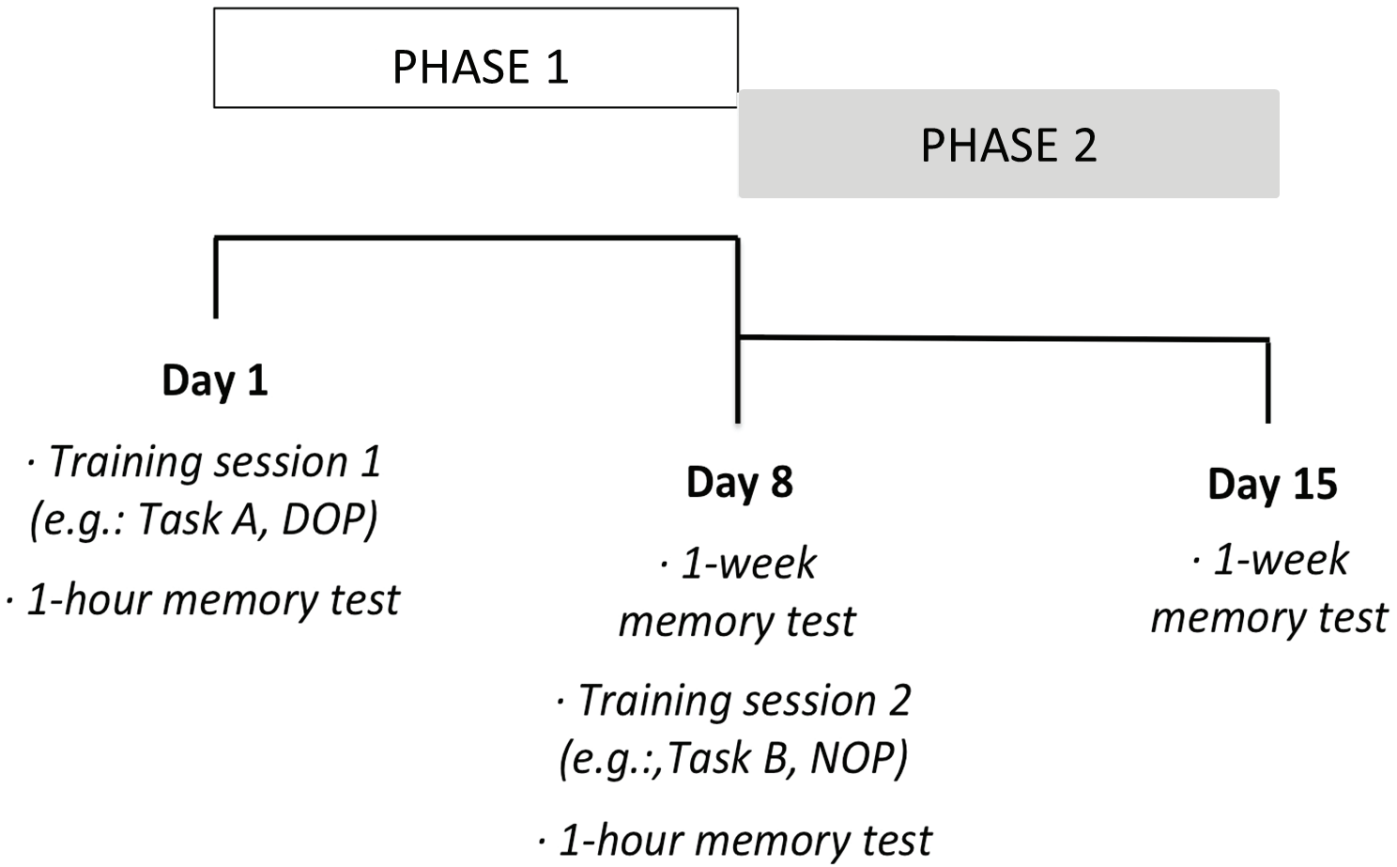
Sequential progression (from left to right) of the two phases of the study.

**Phase 2 (follow-up). Long-term memory test.** The long-term recognition memory task was administered 1 h and 1 week after participants completed each of the training phase (Phase 1) conditions, differential and non-differential. Figure 2 depicts the sequential progression (from left to right) of the two phases of the study. The long-term memory test consisted of the successive presentation of eight photographs of daily objects on the computer screen (the two sample stimuli used in the training condition and six completely new pictures). Participants were required to make an old/new judgment.

### Statistical Analysis

Percentage of correct responses and median correct reaction times obtained for each participant, for each experimental condition, were submitted to a 2 × 2 × 2 mixed analysis of variance with Group (AD and HC) as the between-subjects factor, and Outcomes (differential and non-differential) and Delay (2 and 15 s) as the within-subjects factors. One HC participant, who performed below chance level in both experimental conditions, was excluded from the analyses.

In the long-term memory test, percentage of hits (correctly identified trained objects), percentage of false alarm (“yes” responses to non-trained objects) and the discriminability score (d′ = Z hits rate-Z false alarms rate) (MacMillan and Creelman, 1991; Russo et al., 2017) were submitted to a 2 × 2 × 2 mixed ANOVA with Group (AD and HC) as the between-subjects factor, and Condition (DOP and NOP) and Session (1 h and 1 week) as the within-subjects factors. Two AD patients and one HC participant did not complete one of the tests in one of the conditions—differential versus non-differential; therefore, their data were excluded from this follow-up analysis.

## Results

### Accuracy Analysis

**Figure 3:**
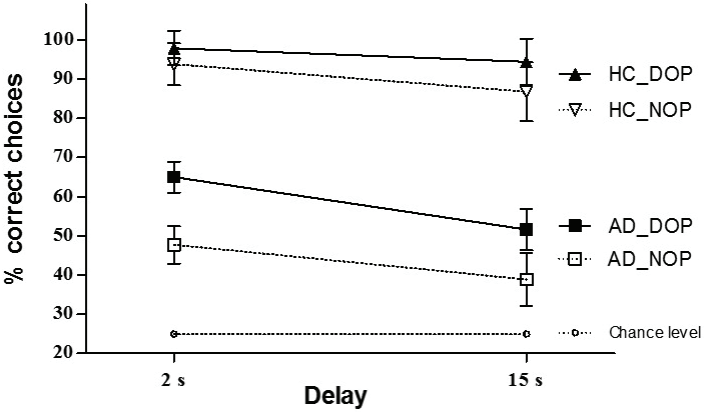
Mean percentage of correct choices for healthy controls (HCs) and Alzheimer’s disease (AD) patients at 2 and 15 s delays under differential (DOP) and non-differential outcomes conditions (NOP). Error bars show the standard error of the mean.

The analysis of correct responses (see Figure 3) showed significant main effects of Group [*F*(1,16) = 39.45, *p* < 0.001, $\eta_{p}^{2}$ = 0.71], Outcomes [*F*(1,16) = 9.19, *p* = 0.008, $\eta_{p}^{2}$ = 0.36], and Delay [*F*(1,16) = 17.48, *p* < 0.001, $\eta_{p}^{2}$ = 0.52]. Overall, AD patients were less accurate than HCs (51 vs. 93% accuracy, respectively). Also, performance was overall better in the DOP condition relative to the NOP condition (77 vs. 67% accuracy, respectively). Finally, overall performance was worse in the long (15 s) than in the short (2 s) delay (68 vs. 76% accuracy, respectively) condition. None of the interactions reached statistical significance (*p* > 0.05).

The order of the training condition (whether differential or non-differential outcomes were arranged in the first session) did not influence the results; thus, as in previous studies, it has not been included in the analyses (Estévez et al., 2003; Plaza et al., 2012).

### Reaction Times Analysis

The analysis of latency data revealed only a significant main effect of Group [*F*(1,16) = 39.5, *p* < 0.001, $\eta_{p}^{2}$ = 0.71]. That is, the HC group (2,169 ms) was overall faster than the AD group (4,765 ms). No other effects or their interactions reached statistical significance (*p* > 0.05).

### Long-Term Memory Test Analysis

The analysis of *hits* (see Table 2) showed significant main effects of Outcomes [*F*(1,14) = 5.74, *p* = 0.031, $\eta_{p}^{2}$ = 0.29] and Group [*F*(1,14) = 91,89, *p* < 0.001, $\eta_{p}^{2}$ = 0.87]. Participants had overall better long-term recognition memory of the trained objects (i.e., more hits) when differential outcomes were arranged during the training Phase 1 (69%) as compared to when they were trained under the NOP (56%); and the AD group showed a lower percentage of hits (32%) relative to the HC group (92%). The following interactions were also statistically significant: Outcomes × Group [*F*(1,14) = 8.97, *p* = 0.01, $\eta_{p}^{2}$ = 0.39] and Session × Outcomes × Group [*F*(1,14) = 5.49, *p* = 0.035, $\eta_{p}^{2}$ = 0.28]. The analysis of the three-way interaction revealed a significant Session × Outcomes interaction for the AD group [*F*(1,7) = 4.20, *p* = 0.05, $\eta_{p}^{2}$ = 0.38] but not for the HC group [*F*(1,7) = 0.10, *p* = 0.69, $\eta_{p}^{2}$ = 0.02]. The interaction was due to a lower retention for the trained objects at the 1-week than at the 1-h memory test (33 vs. 6% of hits, respectively) only under the NOP condition [*F*(1,7) = 7.00, *p* = 0.033, $\eta_{p}^{2}$ = 0.50]. Memory retention did not significantly differ between 1-h and 1-week sessions (50 vs. 44% of hits, respectively) for AD patients in the DOP condition [*F*(1,7) = 0.18, *p* = 0.69, $\eta_{p}^{2}$ = 0.03].

**Table 2:** Mean percentage of hits and false alarms (FA) and d′ score for each group (AD and HC) and outcomes condition (DOP and NOP) in the 1-h and 1-week long-term memory tests (SD in brackets).

	DOP			NOP		
	Hits	FA	d′	Hits	FA	d′
**1-h test**
AD	50% (35)	19% (21)	1.44 (3)	33% (25)	17% (22)	−0.37 (2)
HC	94% (17)	0% (0)	5.79 (1)	88% (23)	0% (0)	5.41 (1)
**1-week test**
AD	44% (17)	23% (28)	1.02 (1)	6% (17)	50% (28)	−2.50 (0,5)
HC	88% (23)	4% (11)	5.08 (2)	100% (0)	0% (0)	6.18 (0)

The analysis of false alarms (see Table 2) yielded significant main effects of Group [*F*(1,14) = 21.81, *p* < 0.001, $\eta_{p}^{2}$ = 0.61] and Session [*F*(1,14) = 10.85, *p* = 0.005, $\eta_{p}^{2}$ = 0.44]. Overall, AD patients had more false alarms than HC (28 vs. 1%, respectively); and false alarms were lower in the 1-h than in the 1-week test (1 vs. 19% false alarms, respectively). The interactions Session x Group and Session × Outcomes × Group were also statistically significant [*F*(1,14) = 6.80, *p* = 0.021, $\eta_{p}^{2}$ = 0.44 and *F*(1,14) = 6.10, *p* = 0.03, $\eta_{p}^{2}$ = 0.30, respectively]. The analysis of the three-way interaction revealed again a significant Session × Outcome interaction only in the AD group [*F*(1,7) = 5.14, *p* = 0.05, $\eta_{p}^{2}$ = 0.42 and *F*(1,7) = 1, *p* = 0.35, $\eta_{p}^{2}$ = 0.12, for the AD patients and for the HC group, respectively]. That is, in the AD group, there was a significant increase of false alarms in the 1-week relative to the 1-h test (50 and 19%, respectively) for the NOP condition [*F*(1,7) = 10.24, *p* = 0.015, $\eta_{p}^{2}$ = 0.60]; while there were no significant differences between the 1-h and 1-week sessions (19 and 23%, respectively) in the DOP condition [*F*(1,7) = 0.47, *p* = 0.52, $\eta_{p}^{2}$ = 0.06].

The analysis of d′ score (see Table 2) showed significant main effects of Group [*F*(1,14) = 75.85, *p* < 0.001, $\eta_{p}^{2}$ = 0.84] and Outcomes [*F*(1,14) = 4.70, *p* = 0.048, $\eta_{p}^{2}$ = 0.25]. Overall, AD patients showed a smaller d′ (0.001) than HCs (5.61); and d′ was higher in the DOP than in the NOP condition (3.35 vs. 2.26, respectively). As in the previous analysis, there were also significant Outcomes by Group [*F*(1,14) = 6.80, *p* = 0.012, $\eta_{p}^{2}$ = 0.37] and Session by Group by Outcomes [*F*(1,14) = 5.75, *p* = 0.031, $\eta_{p}^{2}$ = 0.29] interactions. The analysis of the three-way interaction revealed a significant Session × Group interaction only in the AD group [*F*(1,7) = 7.5, *p* = 0.03, $\eta_{p}^{2}$ = 0.52 and *F*(1,7) = 2.3, *p* = 0.18, $\eta_{p}^{2}$ = 0.25 for the AD patients and for the HC group, respectively]. That is, in the AD group, there was a significant decrease in the discriminability index (d′) from the 1-h to the 1-week session in the NOP condition [*F*(1,7) = 9.76, *p* = 0.017, $\eta_{p}^{2}$ = 0.58], whereas there were no significant differences between the session in the DOP condition [*F*(1,7) = 0.15, *p* = 0.71, $\eta_{p}^{2}$ = 0.02].

## Discussion

The main aim of this study was to investigate if visual memory could be improved with the DOP in AD patients. In order to do so, we used a delayed matching-to-sample task under two experimental conditions: differential (each to-be-remembered visual stimulus was associated with a unique outcome) and non-differential (outcomes were randomly administered following correct responses). As we hypothesized, the DOP improved the delayed recognition of visual information of all participants including AD patients during the training phase. It is worth noting that although the interaction between Outcomes and Group was not significant and the overall performance of the patients did not reach the same levels of the control group, they showed a remarkable significant improvement with only one training session consisting of 36 trials when the DOP was applied as compared to the NOP (15 points vs. 6 points in the AD and the HC groups, respectively). Although the finding of improved memory performance in AD adds to the existing evidence, the most novel finding is the long-lasting memory retention benefit with the DOP training in AD. That is, while we observed the expected memory decline (i.e., less hits, more false alarms, and a lower discriminability) at the 1-week follow-up in the AD patients when trained under the NOP, memory retention (hits, false alarms, and d’) for the same group when trained with the DOP remained unchanged at the 1-week follow-up. Thus, we can conclude that the DOP improved both performance during training and long-term memory retention in AD patients.

The group of HC participants also benefited from receiving the DOP during the training phase, but this positive effect was no longer observed at the follow-up sessions. In fact, their overall performance was very high at both sessions, indicating a possible ceiling effect. Previous studies have found no advantages of the DOP when the task used was too easy to perform (e.g., Estévez et al., 2001; Plaza et al., 2011, 2012), which might also explain the present results. It is also worth noting that a higher DOP effect has been found in long delays, relative to short delays, in discriminative learning with animal subjects (e.g., Brodigan and Peterson, 1976; Peterson and Trapold, 1980), although this finding has usually not been observed in human participants using recognition tasks (Plaza et al., 2011, 2012; Esteban et al., 2014a,b). Our study replicates the latter finding with humans, that is, the DOP effect was not modulated by the delay interval.

Although we did not investigate the neural mechanisms underlying the present effects, we believe that our findings can be best explained in reference to current neurocognitive theories of AD, memory, and learning. With regard to AD, it is generally agreed that neurofibrillary tangles that originate in the anterior subhippocampal cortex and then extend to the hippocampus are the most characteristic neural expression of the disease (Didic et al., 2013). There is also evidence to support that familiarity-based “context-free” VRM and its long-term retention depend on the anterior subhippocampal cortex (Didic et al., 2013). Thus, these two converging lines of evidence may account for the observed worst overall recognition and retention memory performance of AD patients in the NOP relative to the DOP condition in this study. According to the *two-memory system model* (e.g., Savage, 2001; Savage et al., 2004; Ramírez and Savage, 2007; Savage and Ramos, 2009), retrospective memory, which involves the hippocampus, is the only source of information when the NOP is employed (in animals, Savage et al., 2004; in humans, Mok et al., 2009). On the other hand, the prospective memory of the upcoming reward is activated in the DOP, a process that does not rely on the hippocampus. We propose that our finding of improved visual memory in AD only with the DOP fits well with this model. Future studies should directly test this hypothesis by investigating the neurobiological mechanisms underpinning the beneficial effect of the DOP in AD.

To conclude, the present findings add to those reported by López-Crespo et al. (2009) and by Plaza et al. (2012) and support that a simple manipulation of the outcomes arrangement can powerfully affect the memory processes that are altered in healthy aging and dementia. A limitation of this study is the small sample size so that further studies with larger samples calculated *a priori* are needed to explore the usefulness of this procedure as a cognitive intervention technique to enhance short- and long-term visual memory in different clinical populations. Further research is also needed to gain insights into optimal DOP training to obtain long-lasting memory benefits and test whether these learned skills can transfer to real-life situations. The findings of these lines of research could have a significant impact on AD patients’ everyday life such as better recognition of family members by training face-name associations and better medication adherence by training medication-intake characteristics associations for instance.

## Author Contributions

IC was responsible for writing the manuscript and data collection and contributed to the design of the study, statistical analysis, and data interpretation. AV participated in statistical analysis, contributed to data interpretation, and assisted with writing the manuscript. AE was responsible for the design of the study and statistical analysis, contributed to data interpretation, and assisted with writing the manuscript.

All authors approved the final version of the manuscript for submission.

### Conflict of Interest Statement

The authors declare that the research was conducted in the absence of any commercial or financial relationships that could be construed as a potential conflict of interest.
